# UVA/UVB Irradiation Exerts a Distinct Phototoxic Effect on Human Keratinocytes Compared to Human Malignant Melanoma Cells

**DOI:** 10.3390/life13051144

**Published:** 2023-05-08

**Authors:** Otilia Gag, Ștefania Dinu, Horațiu Manea, Iasmina Marcovici, Iulia Pînzaru, Ramona Popovici, Zorin Crăiniceanu, Zsolt Gyori, Gheorghe Iovănescu, Sorin Chiriac

**Affiliations:** 1 Faculty of Dental Medicine, “Victor Babes” University of Medicine and Pharmacy Timisoara, Eftimie Murgu Square No.2, 300041 Timisoara, Romania; 2 Faculty of Medicine, “Victor Babes” University of Medicine and Pharmacy Timisoara, Eftimie Murgu Square No.2, 300041 Timisoara, Romania; 3 Faculty of Pharmacy, “Victor Babes” University of Medicine and Pharmacy Timisoara, Eftimie Murgu Square No.2, 300041 Timisoara, Romania; 4 Research Center for Pharmaco-Toxicological Evaluations, Faculty of Pharmacy, “Victor Babes” University of Medicine and Pharmacy Timisoara, Eftimie Murgu Square No.2, 300041 Timisoara, Romania

**Keywords:** UV radiation, phototoxicity, keratinocytes, skin cancer, malignant melanoma

## Abstract

Solar ultraviolet radiation (UVR) is responsible for the development of many skin diseases, including malignant melanoma (MM). This study assessed the phototoxic effects of UVA, and UVB radiations on healthy and pathologic skin cells by evaluating the behavior of human keratinocytes (HaCaT) and MM cells (A375) at 24 h post-irradiation. The main results showed that UVA 10 J/cm^2^ exerted no cytotoxicity on HaCaT and A375 cells, while UVB 0.5 J/cm^2^ significantly reduced cell viability and confluence, induced cell shrinkage and rounding, generated nuclear and F-actin condensation, and induced apoptosis by modulating the expressions of Bax and Bcl-2. The association of UVA 10 J/cm^2^ with UVB 0.5 J/cm^2^ (UVA/UVB) induced the highest cytotoxicity in both cell lines (viability < 40%). However, the morphological changes were different—HaCaT cells showed signs of necrosis, while in A375 nuclear polarization and expulsion from the cells were observed, features that indicate enucleation. By unraveling the impact of different UVR treatments on the behavior of normal and cancer skin cells and describing enucleation as a novel process involved in the cytotoxicity of UVA/UVB irradiation, these findings bridge the gap between the current and the future status of research in the field.

## 1. Introduction

Being positioned at the external boundary of the body, the skin represents the main barrier conferring protection against chemical, physical and microbiological insults [1,2]. One of the main environmental factors disturbing skin physiology [1], solar ultraviolet radiation (UVR), has been found to generate both morphological and ultrastructural changes in the human epidermis in vivo [3]. Solar UVR comprises three radiation bands, namely UVA (315–400 nm), UVB (280–315 nm), and UVC (200–290 nm). Yet, the one reaching the Earth’s surface is a combination of UVA and UVB lights, since all of the UVC radiation is absorbed by the stratospheric ozone layer [1,3]. UVA is much more abundant than UVB in sunlight, accounting for approximately 95% of the total solar UVR [4]. However, the UVA-to-UVB ratio and the exposure conditions might vary depending on some factors such as the ozone layer, latitude, season, and hour [1]. Some molecules (e.g., DNA [5], proteins [6]) situated within the skin layers can interact with UVR, resulting in pathological implications [7]. Thus, several skin disorders such as erythema, photo-aging, photo-immunosuppression, inflammation, and skin cancers, were linked to UVR exposure [1,2,5].

Malignant melanoma (MM) represents the rarest and the most aggressive skin cancer type, accounting for the majority of deaths related to cutaneous cancers and possessing a rising incidence [8,9]. MM develops through the malignant transformation of melanin-synthetizing cells—the melanocytes [10,11]. Previous scientific evidence supports the implication of solar and artificial ultraviolet light in skin carcinogenesis [12]; approximately 60 to 70% of cutaneous MM cases are caused by UVR exposure [4]. UVR has been classified as a complete carcinogen, acting as both a tumor initiator owing to its mutagenic potential, and promoter by up-regulating several matrix metalloproteinases, by generating additional UV-induced mutations, and by inducing immunosuppression which facilitates the escape of MM cells from immune surveillance [5,13]. 

MM development implicates both UVA and UVB rays. However, these radiation types possess differential toxic effects resulting from their distinct penetrance rates within the epidermis [14]. UVA rays penetrate deeper through the skin layers and trigger the generation of reactive oxygen species (ROS) which further induces DNA damage. UVB rays, on the other hand, are more superficial since they are predominantly absorbed by the stratum corneum of the epidermis. However, UVB radiations induce direct DNA damage through the production of specific photoproducts—cyclopyrimidine dimers (CPDs) and pyrimidine pyrimidone [(6–4)PPs]—leading to UV-signature mutations [14,15], and thus being considered as more carcinogenic than UVA radiations [16]. 

Despite the existing evidence on the damaging effects of UVR on normal skin cells and its role in MM pathogenesis, the data comparing the response of healthy and cancerous skin cells to the individual and the combination UVA and UVB treatments, respectively, remain scarce, limiting the current knowledge regarding the influence of UVR on skin health and disease. Driven by this aspect and aiming to provide a deeper understanding of the damaging effects of UVR at the cutaneous level and how normal and pathological cells behave post-irradiation, this in vitro study comparatively assessed the phototoxic potential of three UVR treatments (UVA, UVB, and UVA/UVB) on HaCaT human immortalized keratinocytes and A375 MM cells.

## 2. Materials and Methods

### 2.1. Reagents and Equipment

Trypsin-EDTA solution, phosphate saline buffer (PBS), dimethyl sulfoxide (DMSO), fetal calf serum (FCS), penicillin/streptomycin solution, Triton X-100, DAPI, and MTT [3-(4,5-dimethylthiazol2-yl)-2,5-diphenyltetrazolium bromide] reagent were purchased from Sigma Aldrich, Merck KgaA (Darmstadt, Germany). Dulbecco’s Minimum Essential Medium (DMEM; 30-2002™) was provided by ATCC (American Type Cell Collection, Lomianki, Poland). Texas Red™-X Phalloidin was provided by Thermo Fisher Scientific Inc. (Waltham, MA, USA). The RealTime-Glo™ Annexin V Apoptosis and Necrosis Kit was provided by Promega (Madison, WI, USA). Cytation 1 and Cytation 5 instruments, as well as the Gen5™ Microplate Data Collection and Analysis Software (Version 3.12), were bought from Biotek Instruments Inc. (Winooski, VT, USA).

### 2.2. Cell Lines and Cell Culture

HaCaT (300493) and A375 (CRL-1619™) cell lines were purchased as frozen vials from CLS Cell Lines Service GmbH (Eppelheim, Germany) and ATCC (American Type Cell Collection, Lomianki, Poland), respectively. The cells were cultured in their specific media—DMEM supplemented with 10% FCS and 1% penicillin (100 U/mL)-streptomycin (100 µg/mL) mixture. The cells were grown in an incubator at 37 °C, and 5% CO_2_, presenting normal morphology and proliferation during all experiments. 

### 2.3. Cell Irradiation with UVA, UVB, and UVA/UVB 

For the irradiation protocol, HaCaT and A375 cells were seeded in 6-, 12- or 96-well plates and exposed to UVA 10 J/cm^2^, UVB 0.5 J/cm^2^, and UVA 10 J/cm^2^/UVB 0.5 J/cm^2^ (treatments that are simply mentioned throughout the manuscript as UVA, UVB, and UVA/UVB) using the Biospectra system (Vilber Lourmat, France). To avoid any interference between the culture media and UVR, as well as to avert the generation of toxic photoproducts upon UVR exposure, the media was changed with PBS following a washing step with the same saline solution. After irradiation, PBS was changed with culture media, and the cells were further incubated at 37 °C, and 5% CO_2_ for 24 h. The non-irradiated cells (referred to in the paper as -UVA, -UVB, and -UVA/UVB) were kept in the same conditions as irradiated cells (in PBS, at room temperature, within the laminar flow, and at the exposure time for each treatment [17]) but were not exposed to UVA, UVB, or UVA/UVB.

### 2.4. Cell Viability Assessment

To assess the viability of HaCaT and A375 cells following UVA, UVB, and UVA/UVB treatment, the MTT protocol was applied after 24 h. In brief, the protocol contained the following steps: (i) cells’ seeding in 96-well plates; (ii) cells’ exposure to UVR and incubation for 24 h at 37 °C and 5% CO_2_; (iii) addition of 100 µL fresh culture media and 10 µL MTT reagent; (iv) 3 h incubation of the plates at 37 °C; (v) addition of 100 µL solubilization solution and incubation of the plates at room temperature for 30 min; and (vi) absorbance reading at 570 and 630 nm using Cytation 5. 

### 2.5. Evaluation of Cell Morphology and Confluence

To verify the impact of UVR on the morphology and confluence of HaCaT and A375 cells, a microscopic evaluation was performed. The cells were cultured in 12-well plates and photographed under bright field illumination at 24 h after UVR exposure using Cytation 1. Image processing was performed using the Gen5™ Microplate Data Collection and Analysis Software (Version 3.12). 

### 2.6. Immunofluorescence Staining of Cellular Components

For the immunofluorescent visualization of the cellular components (cell nuclei, F-actin filaments), HaCaT and A375 cells were seeded in 12-well plates and exposed to UVA, UVB, and UVA/UVB. After 24 h, the cells were washed with ice-cold PBS, fixed with paraformaldehyde 4%, and permeabilized with Triton X/PBS 2%, followed by a blocking step with 30% FBS in 0.01% Triton X. The F-actin fibers were stained with Texas Red™-X Phalloidin (20 min incubation at room temperature). The cell nuclei were counterstained with DAPI (10 min at room temperature). The images were captured using Cytation 1 and processed on Gen5™ Microplate Data Collection and Analysis Software (Version 3.12). 

### 2.7. RT-qPCR Analysis

The total RNA was extracted using the Quick-RNA Miniprep Kit (Zymo Research, Irvine, CA, USA) according to the manufacturer’s protocol and measured using a DS-11 spectrophotometer (DeNovix, Wilmington, DE, USA). For reverse transcription, the Maxima^®^ First Strand cDNA Synthesis Kit (Thermo Fisher Scientific, Inc., Waltham, MA, USA) was applied, and the samples were subsequently incubated into the Tadvanced Biometra Product line (Analytik Jena AG, Göttingen, Germany). The used thermal program was 10 min at 25 °C, 15 min at 50 °C and 5 min at 85 °C. The quantitative real-time PCR was conducted using a Quant Studio 5 real-time PCR system (Thermo Fisher Scientific, Inc., Waltham, MA, USA). The analysis was performed using 20 µL reactions containing pure water, Power SYBR-Green PCR Master Mix (Thermo Fisher Scientific, Inc., Waltham, MA, USA), primers (forward and reverse) and samples’ cDNA. The used primer pairs (provided by Eurogentec, Seraing, Belgium, and Invitrogen) are presented in Table 1. The normalized, relative fold expression data were calculated by applying the comparative threshold cycle (2^−∆∆Ct^) method.

### 2.8. RealTime-Glo™ Annexin V Apoptosis and Necrosis Assay

This assay was performed according to the manufacturer’s recommendations. Briefly, HaCaT and A375 cells were seeded in solid white bottom 96-well plates (Costar^®^ 3917) and exposed to UVA/UVB. Then, 100 µL of culture media and 100 µL of the prepared 2X Detection Reagent (containing Annexin V NanoBiT™ Substrate, CaCl_2_, Necrosis Detection Reagent, Annexin V-SmBiT, and Annexin V-LgBiT) were added to each well, the plates were shaken at 500 rpm for 30 s, and the luminescence and fluorescence (485 ± 20 nm excitation, and 525 ± 30 nm emission) were read on Cytation 5 (at 3 h, 6 h, and 24 h post-irradiation). The background luminescence and fluorescence signals (from no-cell wells) were subtracted from the wells containing cells.

### 2.9. Statistical Analysis

The results are expressed as means ± the standard deviation (SD) of three experiments performed in triplicate. The difference between means was compared by applying the unpaired *t*-test (GraphPad Prism version 9.2.0 for Windows, GraphPad Software, San Diego, CA, USA). The statistically significant differences between the specific controls (non-irradiated cells: -UVA, -UVB, or -UVA/UVB) and the irradiated groups (+UVA, +UVB, or +UVA/UVB) are marked with * (* *p* < 0.05; ** *p* < 0.01; *** *p* < 0.001; **** *p* < 0.0001).

## 3. Results

### 3.1. Cell Viability Assessment

**Figure 1:**
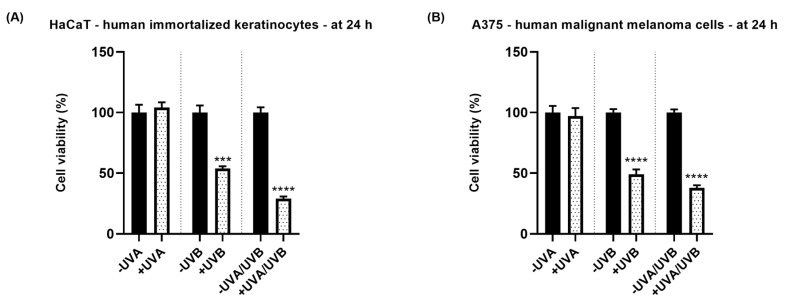
Impact of UVA, UVB, and UVA/UVB on the viability of HaCaT (Panel (**A**)) and A375 (Panel (**B**)) cells at 24 h post-irradiation. The radiation doses were 10 J/cm^2^ for UVA, and 0.5 J/cm^2^ for UVB. The data are presented as viability percentages (%) normalized to -UVA, -UVB, or -UVA/UVB which represent non-irradiated cells and are expressed as mean values ± SD of three independent experiments performed in triplicate. The statistical differences between the non-irradiated and the irradiated groups were verified by applying the unpaired *t*-test. * indicates the statistically significant differences between data (*** *p* < 0.001; **** *p* < 0.0001).

To assess whether UVA, UVB, and UVA/UVB treatments affect the viability of HaCaT and A375 cells, an MTT assay was performed. The results indicated a differential toxic effect of UVA, UVB, and UVA/UVB treatments at 24 h post-exposure against these cell lines (Figure 1). UVA radiation induced no significant changes in the percentage of HaCaT (104.08%) and A375 (97.04%) viable cells. However, UVB exposure reduced the cell viability to 54% (HaCaT) and 49% (A375), respectively. The most cytotoxic effect in both cell lines was noticed after their exposure to UVA/UVB: HaCaT—viability of 28.91% and A375—viability of 37.83%. Although the response to UV radiation was similar in both cell lines in terms of cell viability, A375 cells were slightly more sensitive to UVA and UVB radiations, while HaCaT cells were more sensitive to UVA/UVB exposure.

### 3.2. Evaluation of Cell Morphology and Confluence

**Figure 2:**
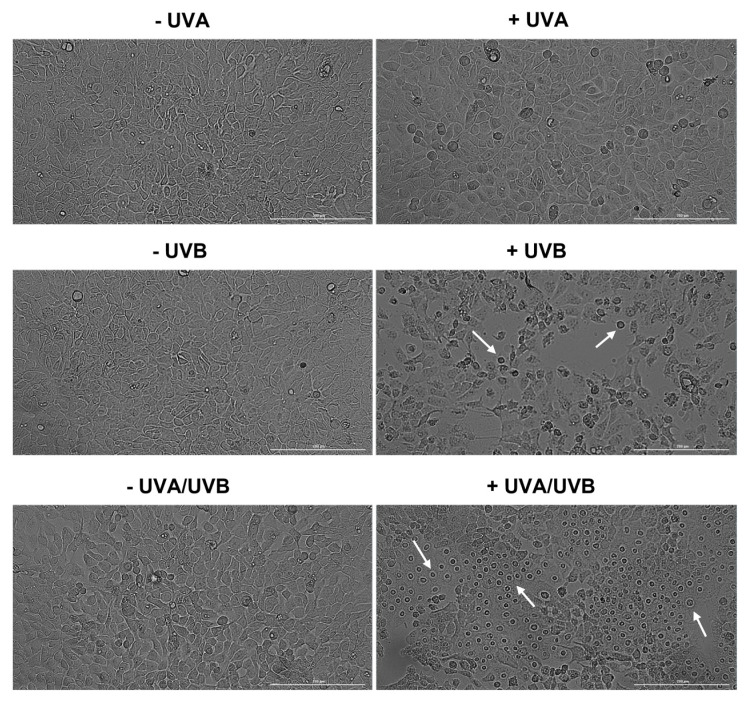
Confluence and morphology evaluation of HaCaT cells at 24 h post-exposure to UVA, UVB, and UVA/UVB compared to non-irradiated cells (-UVA, -UVB, and -UVA/UVB). The radiation doses were 10 J/cm^2^ for UVA and 0.5 J/cm^2^ for UVB. The scale bars indicate 200 µm, and the white arrows show cells presenting abnormal morphology.

**Figure 3:**
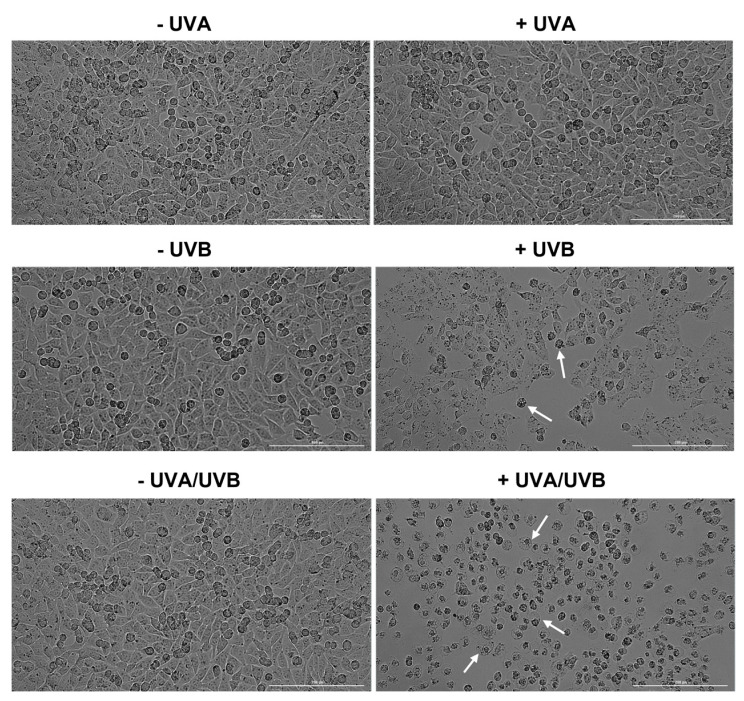
Confluence and morphology evaluation of A375 cells at 24 h post-exposure to UVA, UVB, and UVA/UVB compared to non-irradiated cells (-UVA, -UVB, and -UVA/UVB). The radiation doses were 10 J/cm^2^ for UVA and 0.5 J/cm^2^ for UVB. The scale bars indicate 200 µm, and the white arrows show cells presenting abnormal morphology.

To further assess the phototoxicity of UVA, UVB, and UVA/UVB irradiations, the morphology and confluence of HaCaT and A375 cells were also evaluated. UVA and UVB radiations induced similar morphological changes in HaCaT and A375 cells at 24 h post-irradiation: UVA—no significant shape alterations compared to the respective non-irradiated cells (-UVA) and UVB—visible loss of confluence along with cell shrinkage and rounding compared to the respective non-irradiated cells (-UVB). UVA/UVB treatment, however, exerted a differential effect on the morphology of HaCaT keratinocytes compared to A375 melanoma cells, which is very characteristic of necrotic cell death: the cells are round-shaped, and the nuclei are highly prominent and swollen. In A375 cells, UVA/UVB induced morphological features that are similar to enucleation: cell rounding, polarization, and expulsion of the cell nuclei. These observations are presented in Figure 2 (for HaCaT) and Figure 3 (for A375).

### 3.3. Immunofluorescence Staining of Cell Nuclei and F-Actin 

**Figure 4:**
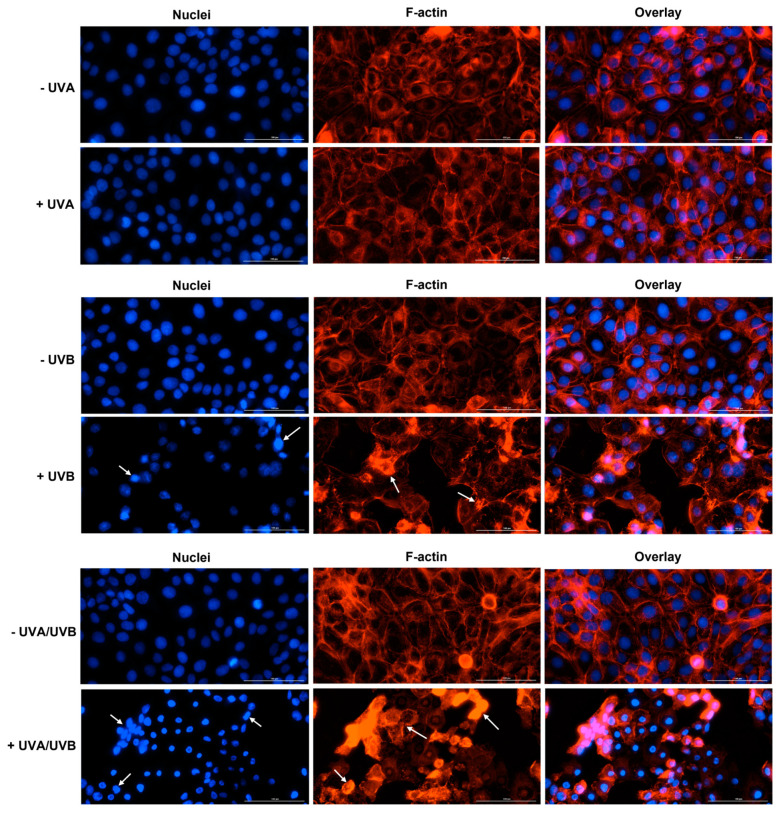
The aspect of cell nuclei and F-actin fibers in HaCaT cells at 24 h post-irradiation with UVA, UVB, and UVA/UVB compared to non-irradiated cells (-UVA, -UVB, and -UVA/UVB). The radiation doses were 10 J/cm^2^ for UVA and 0.5 J/cm^2^ for UVB. The scale bars represent 100 µm and the white arrows indicate an abnormal aspect of cell nuclei and F-actin filaments.

**Figure 5:**
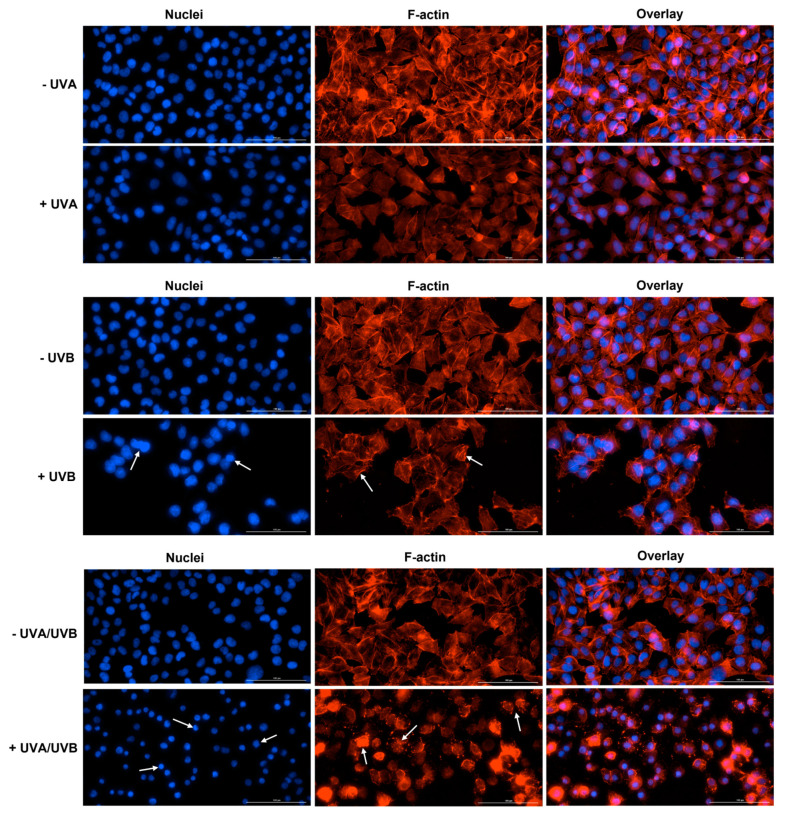
The aspect of cell nuclei and F-actin fibers in A375 cells at 24 h post-irradiation with UVA, UVB, and UVA/UVB compared to non-irradiated cells (-UVA, -UVB, and -UVA/UVB). The radiation doses were 10 J/cm^2^ for UVA and 0.5 J/cm^2^ for UVB. The scale bars represent 100 µm and the white arrows indicate an abnormal aspect of cell nuclei and F-actin filaments.

At 24 h post-irradiation, UVA induced no significant alteration in the aspect of HaCaT or A375 cells’ nuclei or F-actin fibers, compared to control (-UVA)—the nuclei appear round or oval in shape and evenly stained, while the cells maintain their specific shape due to the normal distribution of F-actin in the cell cytoplasm. However, the aspect of nuclei and F-actin was significantly affected by UVB and UVA/UVB irradiations in both cell lines. In HaCaT cells (Figure 4), UVB caused chromatin and F-actin condensation. UVA/UVB led to a visible condensation of chromatin and size reduction of HaCaT cells’ nuclei compared to non-irradiated cells (-UVA/UVB). In addition, this treatment generated a massive aggregation of F-actin bundles in HaCaT cells. In A375 cells (Figure 5), UVB treatment generated visible condensation of chromatin which is observable via an intense staining of the nuclei. The F-actin filaments were also affected by UVB exposure, being destabilized and condensed in the form of stress fibers. UVA/UVB induced condensation and membrane blebbing in A375 cells’ nuclei which also have a smaller size compared to non-irradiated cells (-UVA/UVB). A massive condensation of F-actin fibers that disintegrated into punctate spots can also be observed in A375 cells at 24 h post-irradiation with UVA/UVB. By taking a closer look at the overlay images representing the UVB and UVA/UVB treatments in HaCaT and A375 cells, significant differences in cell morphology and aspect could be discerned. The integrity of the cells seemed preserved following the UVB irradiation, while massive cell destruction was generated by the UVA/UVB exposure.

### 3.4. RT-qPCR Analysis

**Figure 6:**
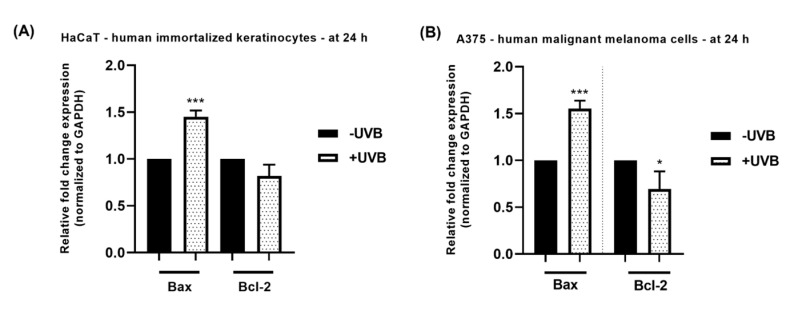
Relative fold mRNA expression of Bax and Bcl-2 markers in HaCaT (Panel (**A**)) and A375 cells (Panel (**B**)) at 24 h post-irradiation with UVB. The mRNA expressions were normalized to GAPDH (used as housekeeping gene). The presented data are mean values ± SD of three independent experiments. The statistical differences between the non-irradiated group (-UVB) and the irradiated group (+UVB) were verified by applying the unpaired *t*-test. * indicates the statistically significant differences between data (* *p* < 0.05; *** *p* < 0.001).

To assess whether the cell death induced by UVB in HaCaT and A375 cells was mediated through apoptosis, the mRNA expression of the pro-apoptotic marker Bax and the anti-apoptotic marker Bcl-2 was determined at 24 h post-irradiation. The results (Figure 6) showed that the UVB treatment increased Bax expression, while decreasing the expression of Bcl-2. The effect was similar in both cell lines, however slightly higher in A375 cells.

### 3.5. RealTime-Glo™ Annexin V Apoptosis and Necrosis Assay

**Figure 7:**
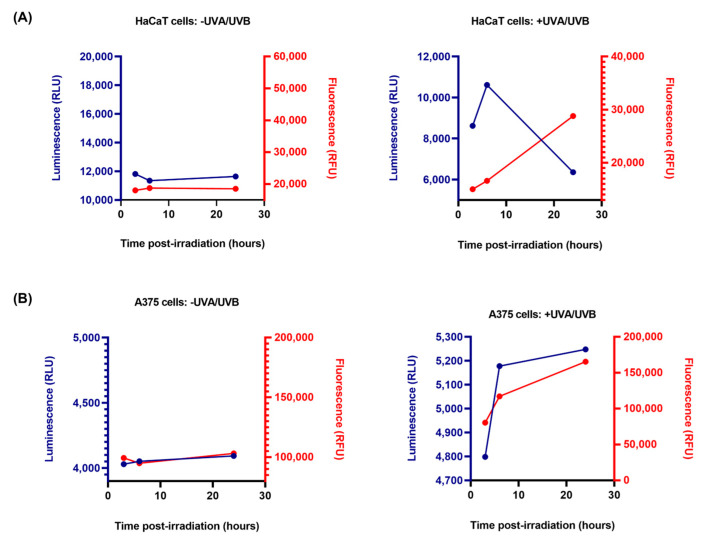
HaCaT (Panel (**A**)) and A375 (Panel (**B**)) cells were treated with the RealTime-Glo™ Annexin V Apoptosis/Necrosis Assay reagent after exposure to UVA/UVB. Luminescence (indicating Phosphatidylserine:Annexin V Binding) and fluorescence (indicating Membrane Integrity) signals were recorded post-irradiation with UVA/UVB at three time points (3 h, 6 h, and 24 h). The -UVA/UVB groups indicate non-irradiated cells. RLU = relative light units; RFU = relative fluorescence units.

To investigate the possible cell death mechanism involved in the cytotoxic effect of UVA/UVA, the RealTime-Glo Annexin V Apoptosis and Necrosis Assay was performed (Figure 7). Three measurements at scheduled time frames were performed, the luminescence (RLU) and fluorescence (RFU) being read post-irradiation with UVA/UVB at 3 h, 6 h, and 24 h. In non-irradiated HaCaT and A375 cells (the -UVA/UVB groups), both luminescence and fluorescence signals remain steady over these time points. However, in the HaCaT and A375 irradiated cells (the +UVA/UVB groups) a distinct variation in the measured signals was observed that are not characteristic to the apoptotic phenotype. In HaCaT cells, the luminescence signal initially increased from 3 h to 6 h, and dropped at 24 h, while the fluorescence signal continuously increased from 3 h to 24 h. In A375 cells, a concomitant increase in both luminescence and fluorescence signals was detected at these time points.

## 4. Discussion

A strong correlation between UVR exposure and the development of a large percentage of cutaneous pathologies has already been established. UVR promotes multiple destructive effects on skin cells such as mutagenesis and carcinogenesis via direct and indirect DNA damage [2], being the major causative factor for skin cancers, MM included [12]. In one of our previous in vitro studies, it has been shown that UVB radiation (at a dose of 40 mJ/cm^2^) proved to be cytotoxic towards both healthy skin cells (HaCaT, 1BR3, HEMa, JB6 Cl 41-5a), and melanoma cells (A375, B164A5) by reducing their viability and inducing cell rounding, floating, shrinkage and detachment [18]. 

The leading hypothesis of this study was that healthy skin cells respond differently to UVA, UVB, and UVA/UVB irradiation compared to cancerous skin cells, thus aiming to assess the in vitro phototoxic effects of these UV treatments on two skin-derived cell lines—HaCaT (human immortalized keratinocytes) and A375 (human malignant melanoma cells). These 2D in vitro models were chosen based on their characteristics. HaCaT cells are spontaneously immortalized keratinocytes of human origin that retain the morphology and functional activity of isolated keratinocytes [19] and were used in this study considering that keratinocytes represent the main defense mechanism of the skin against UVR damage [20]. Another relevant feature of HaCaT cells is that they can organize in a stratified epidermal structure and reconstitute a well-defined epidermis in vivo [19]. The HaCaT cell line was also described as a reliable model for phototoxicity screening [21]. A375 is a human malignant melanoma cell line carrying two mutations (B-RAF and CDKN2, respectively) that were associated with the MM developed from sun-damaged skin [22].

The radiation doses for UVA (10 J/cm^2^) and UVB (0.5 J/cm^2^) were selected based on previous in vitro studies [23,24,25,26,27]. According to Murray et al., the dosage of 100 kJ/m^2^ (or 10 J/cm^2^) UVA is physiologically relevant since it is reached after approximately one hour of sun exposure during summer [24]. In contrast to UVA radiation, UVB doses tested in vitro are significantly lower, largely varying from 0 to 400 mJ/cm^2^ as chosen in some prior studies [25,26,28]. In other reports, however, higher UVB doses were selected (up to 5.6 J/cm^2^) [29,30]. Considering that natural solar radiation consists of both UVA and UVB [31], a combination treatment comprising 10 J/cm^2^ UVA and 0.5 J/cm^2^ UVB was also applied (UVA/UVB). Regarding the time interval at which the experiments were performed (24 h post-exposure to UVA, UVB, and UVA/UVB), in a previous study it has been shown that the damages induced by UV radiation (specifically UVB) are amplified at 24 h post-exposure, while the sensitivity of the assays used to evaluate organelle damage also increases [29].

The main experimental findings of this paper suggest that at 24 h post-irradiation: (i) UVA had no cytotoxic effect on HaCaT and A375 cells in terms of cell viability, morphology, nuclear and cytoskeletal aspect; (ii) UVB exerted significant toxicity which is similar in both HaCaT and A375 cells; (iii) the most cytotoxic effect was observed when the cells were exposed to both UVA and UVB; (iv) UVB exerted a pro-apoptotic effect by modulating the mRNA expression of Bax and Bcl-2, and (v) UVA/UVB induces different phototoxic responses in HaCaT and A375 cells due to the different cell death mechanisms involved.

To assess whether the UVA, UVB, and UVA/UVB treatments affect the viability of HaCaT and A375 cells at 24 h post-irradiation, an MTT assay was performed (Figure 1). The results showed that UVA did not exert any effect on the viability of HaCaT and A375 cells which maintained around the value of 100%, while UVB and UVA/UVB treatments significantly reduced their viability to values < 60% and <40%, respectively. This different response of the cells to direct UVR exposure could be explained by the differential characteristics of UVA and UVB radiations. The first one exerts its toxicity by ROS generation which might have been counteracted by the innate defense mechanisms of the cells, while UVB is more genotoxic by directly affecting cellular DNA [4,9]. Bajgar et al. suggested that the phototoxicity of UVR on epidermal keratinocytes depends on the radiation type and dosage, with higher cell damage being caused by UVB at lower doses compared to UVA [32]. Another study showed a dose-dependent decrease in the viability of immortalized and primary human skin keratinocytes (HaCaT, and HKN cells) recorded at 48 h following their irradiation with UVA at various doses (survival rate of 50% at 12 J/cm^2^ for HaCaT, and 6 J/cm^2^ for HKN) [33]. A large and significant decrease in cell viability was also found in fibroblasts irradiated with UVA at doses of 9 and 15 J/cm^2^, while UVA radiation at doses of 3 and 5 J/cm^2^ did not influence the viability of the cells (24 h post-irradiation) [34]. UVB was found to induce damage in HaCaT keratinocytes at doses starting with 0.52 J/cm^2^ [29], and significantly reduce the survival rate of B16 melanoma cells at doses of 200 and 300 mJ/cm^2^ [35]. The association of UVA with UVB exerted a higher cytotoxic effect compared to the individual treatments, probably because UVA radiation potentiated the cytotoxicity induced by UVB radiation. A recent report also showed that the interaction between UVA and UVB leads to augmented toxicity in vitro against healthy skin cells: the pre-exposure of HaCaT cells to long-wavelength UVA radiation (UVA1) at the dose of 160 kJ/m^2^ enhanced the cell death induced by UVB radiation (doses of 0.15–0.3 kJ/m^2^) [36].

Considering that the morphological characteristics are useful tools that describe cell death [37], the UVR impact on cell morphology was further assessed (Figure 2—HaCaT, and Figure 3—A375). In accordance with the viability results, the morphology and confluence of HaCaT and A375 cells were not affected by UVA exposure. However, at 24 h post-irradiation with UVB, several signs of cytotoxicity were observed in both cell lines such as cell rounding, shrinkage, and loss of confluence. Interestingly, UVA/UVB had a differential phototoxic effect in the irradiated cell lines. Although the cell confluence was not significantly reduced, HaCaT cells showed signs of necrosis (highly prominent, and swollen nuclei [38]), while in A375 cells a significant confluence reduction accompanied by enucleation (indicated by nuclear polarization, and expulsion from the cells) was observed [39]. Apoptosis remains the most extensively studied type of cell death [40], however, other mechanisms might also be involved in the cytotoxic effect exerted by chemical or physical agents. In this study, two different cell death types induced by UVA/UVB irradiation in human skin cells at 24 h post-irradiation were observed: necrosis in HaCaT human immortalized keratinocytes and enucleation in A375 malignant melanoma cells. Necrosis is described as an uncontrolled and drastic type of cell death that is characterized by oncosis (swelling of cell nuclei and organelles), and loss of membrane integrity which consequently leads to the release of intracellular content, and inflammatory response [41]. UVR was previously found to induce necrosis in keratinocytes, along with other cell death types (i.e., apoptosis, autophagy, and pyroptosis) which became targets for preventing UVR-induced cutaneous injury [20]. Enucleation is a process occurring during erythropoiesis, which manifests with several changes occurring at the cellular level such as dramatic condensation of chromatin, nuclear polarization and expulsion, and rearrangement of F-actin bundles which condensate behind the extruding nucleus [40]. To date, little is known about enucleation generated in cancer cells. A previous study by Paunescu et al., however, describes enucleation as a cell death mechanism involved in the anticancer effect of colloidal suspensions of Fe3O4 nanoparticles on two breast cancer cell lines (SK-BR-3 and MCF-7) [42]. However, to the best of our knowledge, this is the first study reporting enucleation as an underlying mechanism of UVR-induced phototoxicity in MM cells. 

To further evaluate the impact of UVRs on the aspect of cellular components, immunofluorescence staining highlighting the cell nuclei, as well as the cytoskeletal F-actin, was performed at 24 h post-irradiation (Figure 4—HaCaT, and Figure 5—A375). Actin is the major cytoskeletal constituent that suffers dramatic changes during different stages of apoptosis, being classified as both sensor and mediator of this cell death type [43,44]. Furthermore, the actin cytoskeleton is also regulated by UV radiation. Recently, UV light was shown to disrupt F-actin filaments in MeWo malignant melanoma cells [45]. In another study, Wang et al. showed that narrow-band UVB irradiation (at a dose of 100 mJ/cm^2^) induced F-actin disassembling in B16 malignant melanoma cells which was observable at 30 min after irradiation, and more evident at 6 h, taking the aspect of punctate spots [35]. Similar findings were reported in this paper in the case of UVB irradiation in both HaCaT and A375 cells: F-actin destabilization and aggregation in stress fibers accompanied by nuclear condensation. UVA/UVB produced massive condensation of chromatin and F-actin in HaCaT cells. In A375 cells, F-actin disintegration into punctate spots, and nuclear condensation were noticed. These observations confirm the phototoxicity of UVB and UVA/UVB in these cell lines. However, a difference could be spotted between the effects of these treatments on the cellular components since the integrity of the HaCaT and A375 cells is significantly affected only by the UVA/UVB irradiation (Figure 4 and Figure 5—overlay images for UVB and UVA/UVB). 

To elucidate the possible mechanisms behind the phototoxic events observed following the HaCaT and A375 cells’ irradiation with UVB, a RT-qPCR analysis was performed. In accordance with the results obtained so far, at 24 h post-irradiation, UVB exerted a similar effect in both irradiated cell lines—a significant increase in the mRNA expression of Bax accompanied by the decrease in the mRNA expression of Bcl-2—which illustrates its pro-apoptotic activity also supported by previous results [29]. 

To detect the possible cell death mechanism induced by UVA/UVB in HaCaT and A375 cells, the RealTime-Glo™ Annexin V Apoptosis and Necrosis Assay was applied (Figure 7). In this assay, apoptotic agents produce a time- and dose-dependent increase in luminescence signal (corresponding to the Annexin V binding to the externalized phosphatidylserine), prior to the temporal increase in fluorescence signal (corresponding to loss of membrane integrity, and secondary necrosis) [46]. The variations observed in HaCaT and A375 cells were not specific to an apoptotic phenotype. In the HaCaT cells irradiated with UVA/UVB, an initial increase in the luminescence signal was observed from 3 h to 6 h, followed by a drop at 24 h. The fluorescence signal in HaCaT cells increased over-time (from 3 h to 24 h). A similar variation, indicating a necroptotic response, was obtained after the treatment of U937 cells with 25 ng/mL TNFα + 10 µM Z-VAD-FMK [47]. These results complete the observations made during the cell morphological evaluation (Figure 2) in which necrotic-like features were detected in HaCaT cells irradiated with UVA/UVB. In A375 cells, a concomitant increase in both luminescence and fluorescence signals was detected, which indicates an alternative cell death mechanism to apoptosis or necrosis. These findings denote that the UVA/UVB exposure differentially affects HaCaT and A375 cells due to distinct mechanisms involved. 

Although these results comparatively illustrated how healthy and malignant cells respond to the phototoxic effects of UVR at 24 h post-irradiation, the reduced number of cell lines used in the experiments and the short time frame post-irradiation at which the UVR-induced toxicity was assessed might constitute potential limitations of the study. A deeper insight into the cutaneous toxicity exerted by UVR could be obtained by increasing the number of evaluated cell lines, by diversifying the types of skin cells used within the study, and also by prolonging the incubation time post-irradiation (over 24 h). 

## 5. Conclusions

In the present study, UVA 10 J/cm^2^ was found to induce no cytotoxic effect at 24 h post-irradiation in HaCaT and A375 cells, while significant cytotoxicity was observed after the cells’ irradiation with UVB at a lower dose (0.5 J/cm^2^). Although both HaCaT and A375 cells responded similarly to the individual UVA and UVB treatments, the UVA/UVB irradiation induced a differential phototoxic effect in these cell lines which showed signs of necrosis and enucleation, respectively. These results bring new insight into the phototoxicity of UVR, and its impact on keratinocyte and melanoma behavior, illustrating the similarities and differences in the response of normal and cancer skin cells to various UVR treatments (UVA, UVB, and UVA/UVB). Based on these findings, further studies should investigate necrosis and enucleation as possible cell death mechanisms involved in UVA/UVB-induced cytotoxicity in cutaneous cells.

## Figures and Tables

**Table 1:** The oligonucleotides of the primers used for the Rt-qPCR analysis.

Primer	Forward	Reverse
GAPDH *	5′ AAG-GTG-AAG-GTC-GGA-GTC-AAC 3′	5′ GGG-GTC-ATT-GAT-GGC-AAC-AAT-A 3′
Bax	5′ GCCGGGTTGTCGCCCTTTT 3′	5′ CCGCTCCCGGAGGAAGTCCA 3′
Bcl-2	5′ CGGGAGATGTCGCCCCTGGT 3′	5′ GCATGCTGGGGCCGTACAGT 3′

* housekeeping gene.

## Data Availability

Not applicable.
